# Neuromyelitis Optica Spectrum Disorder with Tumefactive Demyelination mimicking Multiple Sclerosis: A Rare Case

**DOI:** 10.3389/fneur.2016.00073

**Published:** 2016-05-11

**Authors:** Ujjawal Roy, Dinesh Satyanarayan Saini, Koushik Pan, Alak Pandit, Goutam Ganguly, Ajay Panwar

**Affiliations:** ^1^Department of Neurology, Bangur Institute of Neurosciences, The Institute of Post-Graduate Medical Education and Research, Kolkata, India; ^2^Department of Neurology, King George’s Medical University, Lucknow, India

**Keywords:** neuromyelitis optica spectrum disorder, longitudinally extensive transverse myelitis, aquaporin-4, tumefactive multiple sclerosis, therapeutic plasma exchange

## Abstract

Neuromyelitis optica spectrum disorder (NMOSD) is a diverse condition which not only encompasses isolated longitudinally extensive transverse myelitis (LETM) and optic neuritis but also includes area postrema syndrome, acute brainstem syndrome, symptomatic narcolepsy or acute diencephalic clinical syndrome, and symptomatic cerebral syndrome. Imaging may reveal periependymal lesions surrounding the ventricular system or involvement of corticospinal tracts, area postrema, diencephalon, and corpus callosum. Rarely, there may be hemispheric tumefactive lesions that enhance in a “Cloud-like” fashion on gadolinium injection unlike in tumefactive multiple sclerosis where there is incomplete ring enhancement. Here, we present a case of aquaporin-4 positive relapsing NMOSD who presented to us with recurrent episodes of paraparesis with LETM and tumefactive lesions of brain on imaging, which enhanced in an incomplete ring like pattern resembling multiple sclerosis.

## Background

Neuromyelitis optica (NMO) is an inflammatory CNS disorder that was initially thought to be a variant of multiple sclerosis (MS), but with the discovery of serum antibodies (Ab) against aquaporin-4 (AQP4), it is now considered a distinct entity from MS. Traditionally, NMO was considered a monophasic disorder that consisted of simultaneous bilateral optic neuritis and transverse myelitis, but relapsing cases have been clearly described (1). Magnetic resonance imaging (MRI) reveals involvement of more than three vertebral segments, which is synonymous with longitudinally extensive transverse myelitis (LETM) (1). Nevertheless, some cases may be difficult to diagnose with certainty, especially where there are overlapping features of both NMO and MS and those who test negative for AQP4-Ab. Since NMO is primarily an astrocytopathy and MS is a demyelinating disorder, treatment of these disorders is quite distinct and they need to be differentiated so that appropriate treatment is started as early as possible to reduce the significant morbidity associated with these disorders. Hence, knowledge of lesion location and characterization of these lesions on neuroimaging is of utmost importance before treatment can be initiated (1).

## Case Report

A 21-year-old girl, while attending college, was referred to our outpatient department with a 12-day history of gradual onset of quadriparesis. Weakness was present both proximally and distally, was initially asymmetric to start with but reached its nadir and became almost symmetric within 2 days. Along with her weakness she also experienced decreased sensation up to her lower chest and developed incontinence of urine. There was no history of any convulsions, loss of consciousness/altered sensorium, decreased vision, diplopia, facial weakness, or dysphagia. After review of the initial MRI and cerebrospinal fluid (CSF) profile, the patient had been prescribed anti-tubercular drugs along with steroids 1 week earlier by a local practitioner and associated with mild improvement in her symptoms.

On examination, she had a temperature of 37.2°C; no palpable lymph nodes were detected. The remainder of her general examination was unremarkable. On neurological examination, she was conscious, well-oriented but apprehensive. Tone was increased in both lower limbs; however, it was normal in the upper limbs. Strength was decreased in all four limbs (2/5 in both upper limbs and 1/5 in lower limbs), and muscle stretch reflexes were increased with Babinski sign bilaterally. On sensory examination, superficial and deep sensation to all modalities was diminished below the C7 dermatome.

**Figure 1:**
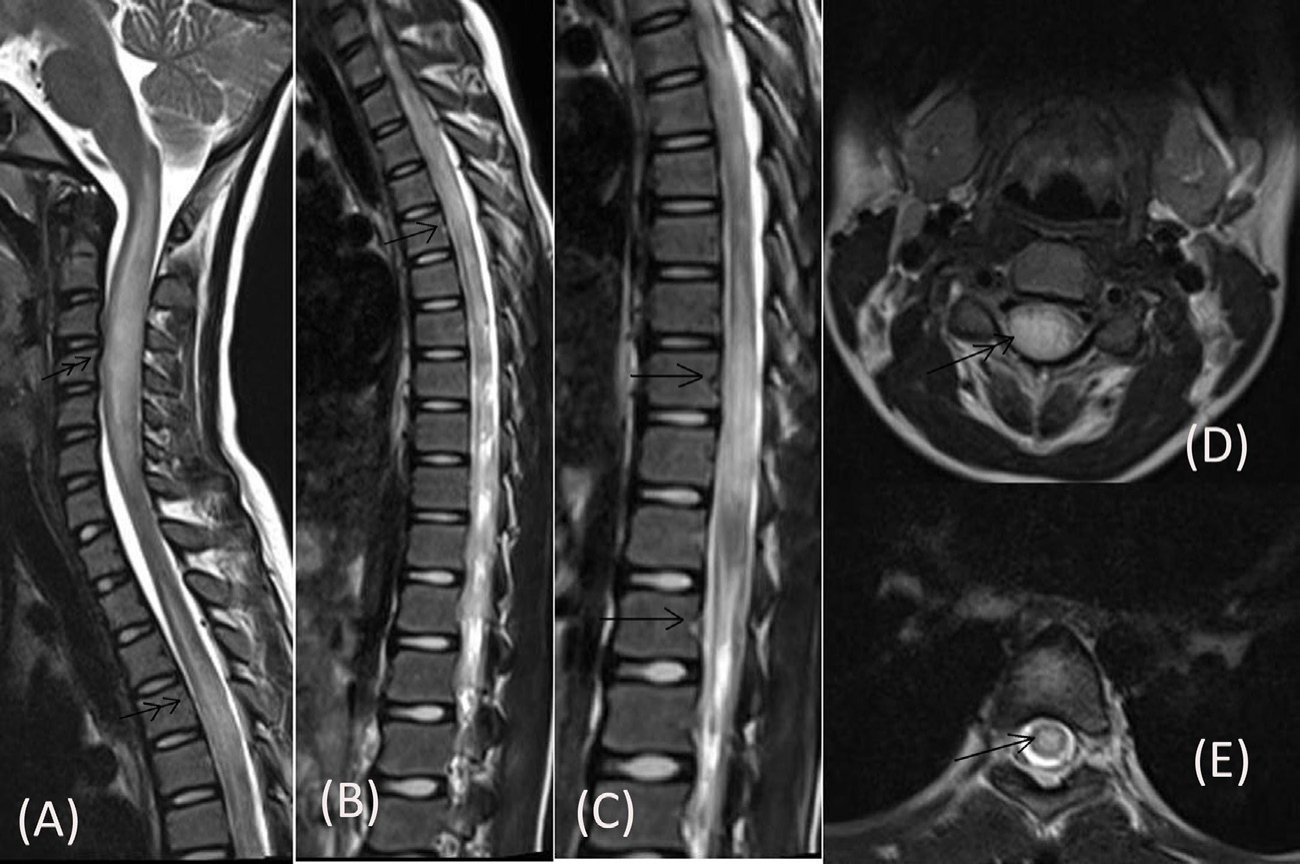
**MRI of cervical spine done prior to admission showing long segment centrally located lesions in form of T2 hyperintensity in cervical cord (double arrow) [Saggital (A), Axial (D)] and dorsal cord (single arrow) [Saggital (B,C), Axial (E)]**.

Routine blood investigations, including complete blood count, erythrocyte sedimentation rate, blood sugar (BS), serum electrolytes, and liver and kidney function tests were normal. Serologies for human immunodeficiency virus, hepatitis C virus, hepatitis B surface antigen, and tuberculosis skin test were negative. Collagen vascular disease work up was also non-contributory. Chest X-ray and ultrasonography of the abdomen were also normal. MRI of the cervical spine, done prior to admission, had revealed long segment lesions with T2 hyperintensity in cervical and dorsal cord favoring demyelination (Figure 1). CSF study done prior to presentation at our hospital had shown 350 cells out of which 62% were neutrophils and 38% were mononuclear cells, protein was 180 mg/dL and sugar was 50 mg/dL (BS: 82).

**Figure 2:**
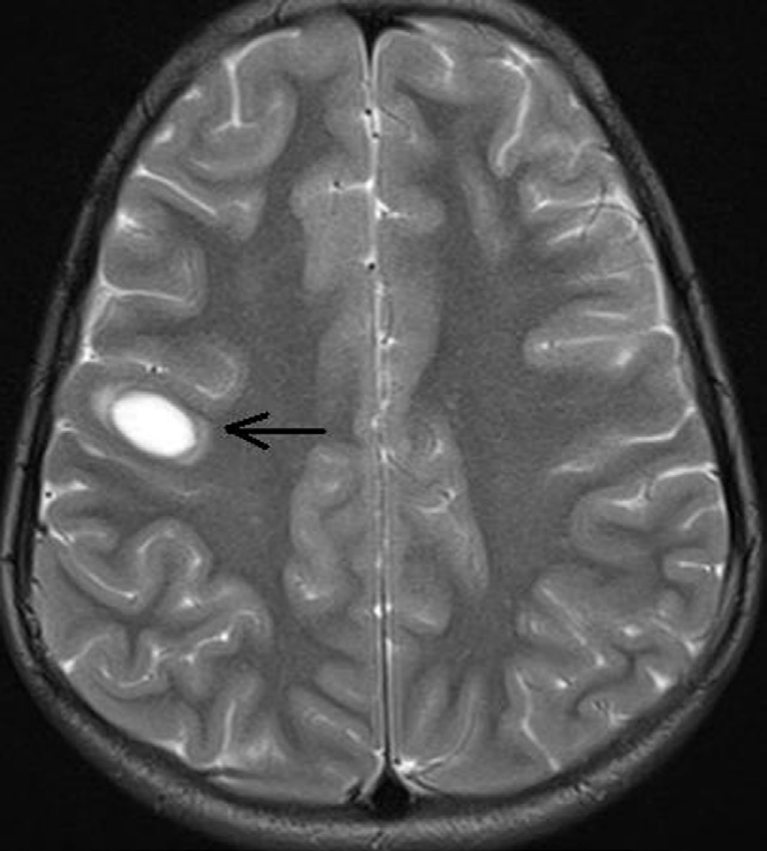
**MRI screening of brain showing single focal well-circumscribed hyperintense lesion (arrow) in T2 sequence at right subcortical frontal region (at gray white interface)**.

On presentation at our center, we performed a MRI of the brain that revealed a single well-circumscribed T2 hyperintense lesion in the right subcortical frontal region (at gray white interface) (Figure 2) without any diffusion restriction or contrast enhancement. Accordingly, we repeated the CSF study in view of these lesions that showed 300 cells out of which 58% were neutrophils and 42% were mononuclear cells; however, there were no red blood cells. Protein was 203 mg/dL, sugar was 60 mg/dL (BS: 82), tuberculosis polymerase chain reaction was negative, and adenosine deaminase came out to be 3.2 U/L. Furthermore, oligoclonal band (OCB) was positive in the CSF. In view of the longitudinally extensive myelitis and a high CSF count, AQP4-Ab was also ordered. Although visual-evoked potentials (VEP) test was normal bilaterally, somatosensory evoked potentials (SSEPs) were suggestive of prolonged latency of N13 and N17 waveforms.

We made the provisional diagnosis of NMO spectrum disorder (NMOSD) in light of overall picture. She received a 5-day course of 500 mg daily intravenous methyl-prednisolone (IVMP) followed by oral prednisolone (OPN) at a dose of 1 mg/kg/day (60 mg). Significant improvement in power was noted (4/5 in upper limbs and 2/5 in lower limbs) at the seventh day from initiation of therapy. Meanwhile AQP4-Ab (done by indirect immunofluorescence) was positive (1:500 titer), so azathioprine therapy was initiated at a dose of 50 mg daily and subsequently increased to 100 mg/day over the next 4 weeks with monitoring of blood counts and liver function tests. Meanwhile, OPN was continued at the same dose.

**Figure 3:**
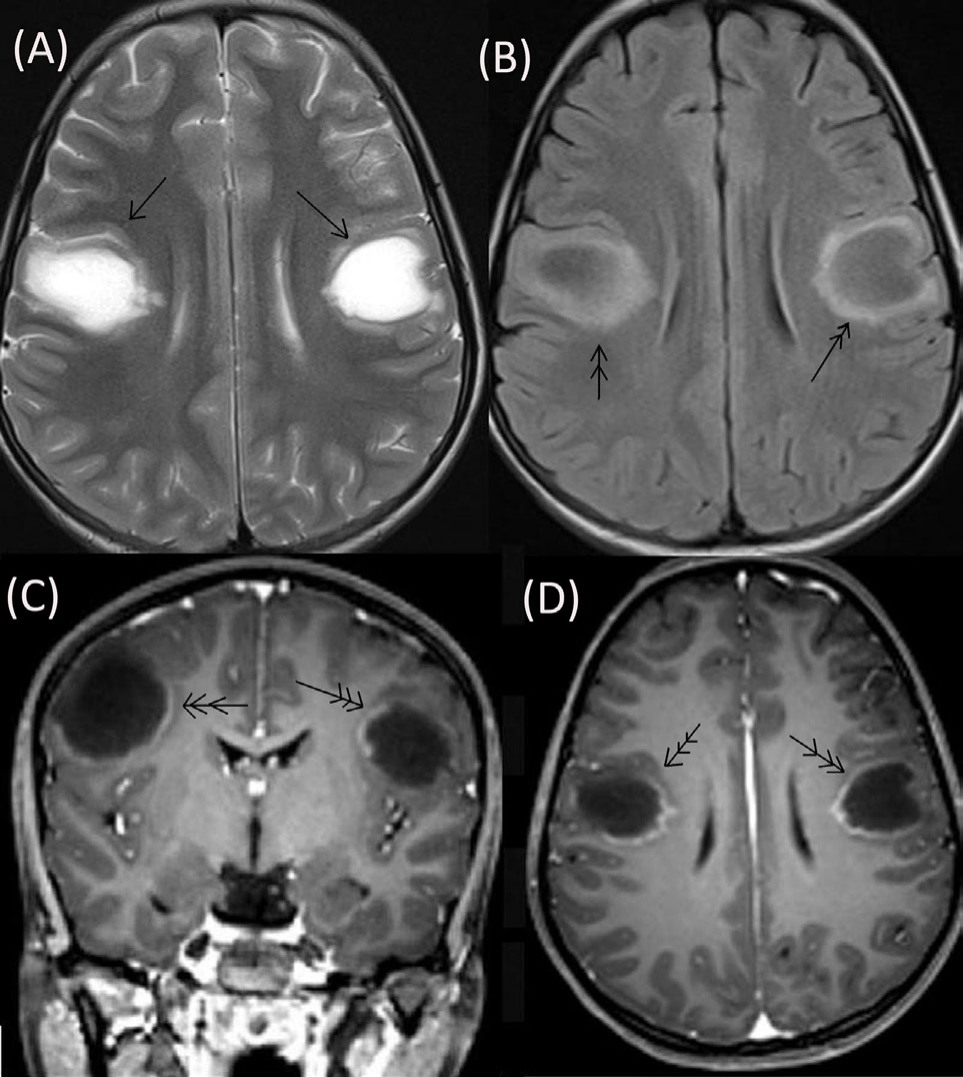
**Repeat MRI brain showing bilateral frontal–parietal hyperintensities on T2 sequence (single arrow) with central hypointensity on FLAIR (double arrow) (A,B)**. On contrast injection incomplete ring enhancement was seen (triple arrow) **(C,D)**.

**Figure 4:**
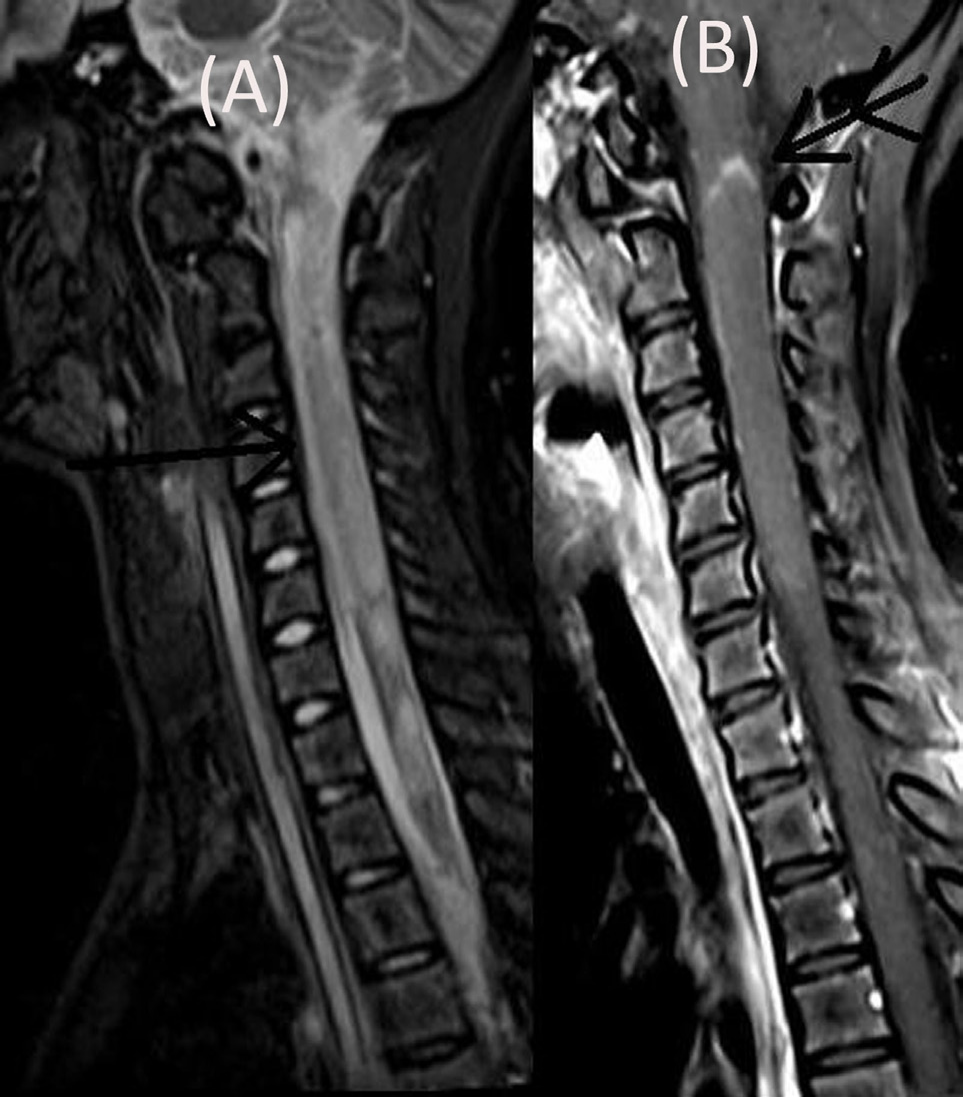
**Cervical MRI showing long segment T2 hyperintensity (arrow) (A) patchily enhancing in cervical cord (double arrow) and dorsal cord (B)**.

The patient’s neurological status remained unchanged over for next 5 weeks when she developed a left focal motor seizure, neck weakness, and bilateral facial and bulbar palsy along with increased weakness in all four limbs. A repeat MRI of the brain revealed bilateral frontal–parietal hyperintensities on T2 sequence with central hypointensity on FLAIR (Figures 3A,B). Furthermore, on contrast injection incomplete ring enhancement was noted (Figures 3C,D). Cervical MRI showed patchy enhancing long TR hyperintensities in cervical and dorsal cord favoring demyelination (Figures 4A,B). We decided to treat the patient with five cycles of therapeutic plasma exchange (TPE) along with IVMP. On the fourth day of TPE, she again started recovering with regard to extremity strength and with significant improvement in facial and bulbar weakness as well. She was discharged on azathioprine and continued physiotherapy at home. At her 4 months follow–up, she was ambulatory, had re-joined her college, and was doing fine otherwise. Subsequently, OPN was tapered off and azathioprine maintained at a dose of 125 mg/day.

## Discussion

Recently, a revised diagnostic criterion for NMOSD has been proposed by the International Panel for NMO Diagnosis that has stratified this disorder further by serologic testing (NMOSD with or without AQP4-IgG positivity). The “core clinical characteristics” have been described as clinical syndromes or MRI findings related to optic nerve, spinal cord, area postrema, other brainstem, diencephalic, or cerebral presentations, and only one out of these six “core clinical characteristics” is required for the diagnosis of NMOSD in patients with AQP4-IgG positivity. Patients who are AQP4-IgG negative must experience two or more different core clinical characteristics either in a single clinical attack or multiple attacks out of which one of the clinical events must be one of the three most common clinical characteristics of NMOSD: optic neuritis, LETM MRI lesion, or an area postrema clinical syndrome (2). Accordingly, we had diagnosed this patient as having AQP4-IgG positive NMOSD after the first attack.

In NMOSD, LETM (more than or equal to three vertebral segment of the spinal cord, mainly involving central/gray matter, appearing as a T1 hypointensity) and optic nerve involvement in the form of long-length/posterior-chiasmal lesions is characteristic. In brain, there are periependymal lesions surrounding the ventricular system (wide-based along the ependymal lining), along with lesions involving corticospinal tracts. Rarely, there may be hemispheric tumefactive lesions that have faint or “Cloud-like” enhancement on MRI because of the preservation of integrity of blood–brain barrier (3). Cheng et al. studied the clinical and radiographic features of NMO patients with extensive brain lesions (EBLs) in China. They included 16 NMO patients with EBLs out of which 4 had tumefactive-like lesions on MRI of the brain. Contrast enhancement was absent or faint in three and open-ring in one among the patients who had tumefactive-like lesions (4). By contrast, patients with MS may also have large demyelinating lesions on MRI, but these lesions characteristically demonstrate incomplete or near complete (ovoid) ring enhancement (5, 6). This presentation of MS is called “tumefactive multiple sclerosis (TMS)” in which a solitary intracranial lesion larger than 2.0 cm in diameter is a classical MRI finding, although multiple lesions have also been reported creating a diagnostic puzzle for both clinicians and radiologists. TMS usually carries a benign prognosis, so distinguishing its lesions from infection, abscess, and especially glioma is of utmost importance in avoiding unnecessary medical or surgical interventions (7). In our case, the patient’s MRI had LETM with bilateral tumefactive lesions of the brain with incomplete ring-like enhancement of the lesions that have been rarely reported in association with NMOSD.

Thus, without the identification of AQP4-Ab positivity, this could have posed a diagnostic dilemma and the patient could have been misdiagnosed as TMS. Furthermore, OCB were positive in the CSF and while the absence of CSF OCBs has been considered as a supportive evidence for NMOSD, they may be transiently detectable at the time of an attack (2). Additionally, there were some features classically described on MRI which favored a diagnosis of MS: MRI in patients with MS can demonstrate short, often multiple, peripheral, asymmetrical, posterior predominant lesions of spinal cord along with the classical “dawson fingers” (perpendicular to ventricles), U-fiber involvement, lesions of the inferior lateral ventricle, and temporal lobe of brain (3). These radiological features are clinically useful as treatment paradigms differ in cases of MS and NMOSD and an incorrect diagnosis poses a chance of significantly increased morbidity in these patients (2). This particularly applies to and concerns severe NMO relapses misdiagnosed as MS and treated with MS-modifying drugs. Exacerbations of NMO have been observed after treatment with interferon β, natalizumab, and fingolimod (3).

Our case had a relapsing type of presentation, and AQP4-IgG was positive in her serum. Researchers have observed that patients with monophasic NMO are more commonly AQP4-IgG-seronegative as compared to those with established relapsing disease (8). Long-term outcome and disability of NMOSD depends on many factors, one of the most important being successful treatment of attacks. Hence, clinical attacks should be treated aggressively and an early and timely escalation of therapy is recommended (9). Researchers have shown that pulse therapy with IVMP significantly improves clinical disability and augments the preservation of the retinal nerve fiber layer thickness (10, 11). When remission is absent or insufficient, five to seven cycles of TPE improves attack-related disability in both seropositive and seronegative patients with the possibility of the existence of other types of auto-Ab (12, 13). In our case, treatment was started at the earliest possible timeframe after presentation (even before AQP4-IgG result was available). Furthermore, in the second attack, we treated the patient with TPE and IVMP in combination and significant improvement was observed over the next few days. This is in contrast with the observations of previous researchers that have shown that presence of EBLs in NMO may portend a worse long-term prognosis (4). To summarize, patients who have EBLs may be candidates for aggressive therapy, which may change the ultimate outcome in these patients.

## Conclusion

Neuromyelitis optica spectrum disorder should be diagnosed promptly so that appropriate treatment is started as early as possible to reduce the significant morbidity associated with this disease. Therefore, knowledge of typical and atypical lesions on neuroimaging is of utmost importance when treating these disorders. Although, tumefaction with incomplete ring enhancement is more commonly observed in MS, it can occur in this disorder and its presence is consistent with NMOSD, especially if other clinical, laboratory, and imaging findings support this diagnosis. Further studies may be required to know the exact incidence, cause, and associations of such presentation on imaging.

## Ethical Statement

This study was carried out in accordance with the recommendations of ethical committee of IPGMER, Kolkata with written informed consent from the patient (Consent: taken).

## Author Contributions

Dr. UR and Dr. DS compiled the data of the case and prepared the manuscript. Dr. KP and Dr. Pandit reviewed the literature. Dr. GG and Dr. Panwar helped in the concluding remarks apart from critically analyzing the paper and the addition of several points in discussion.

## Conflict of Interest Statement

The authors declare that the research was conducted in the absence of any commercial or financial relationships that could be construed as a potential conflict of interest.
